# Scarlet Fever in Horses

**Published:** 1884-01

**Authors:** John C. Peters

**Affiliations:** New York


### THE JOURNAL

OF

### COMPARATIVE MEDICINE AND SURGERY.

VOL. V. JANUARY, 1884. No. 1.

### ORIGINAL COMMUNICATIONS.

### ART. I.—SCARLET FEVER IN HORSES.

BY DR. JOHN C. PETERS, OF NEW YORK.

FIRST ATTEMPT TO FIND THE DISEASE.

SEVERAL years ago I noticed in some French medical journals short accounts of scarlet fever in horses. The disease was spoken of so assuredly that I took it for granted that almost everybody, except myself, was well acquainted with it. I had then been chairman of the Committee on Hygiene of the Medical Society of the County of New York for three years, viz.: just before and during the great movement for better street cleaning, and knew that filth-diseases abounded both in horse and man. Numerous complaints had also come to me from physicians and hospitals of the offensive condition of many stables, public and private, which are so thickly scattered in all parts of the city. I had also been long and deeply interested in the study of the origin and conveyance of all epidemic, infectious, and contagious diseases: hence duty and inclination led me to hunt up scarlet fever and perhaps other infectious and contagious diseases among horses. I quickly found that I had quite a difficult undertaking before me, as I did not want just then to be guided by books or authorities, but wished to look at horse diseases with fresh, if not young eyes. The subject was also rather interesting to a medical man.

There was a smart epidemic of so-called pink-eye then prevailing, also a sprinkling of virulent, foul-air influenza, some mutterings of a recrudescence of the cerebro-spinal, or foul-air rheumatic fever, which so affected man and horse in 1871-2; sore throat and strangles were not rare, and abscesses about the neck, and dropsy of the legs were not uncommon. Somethings looked to me marvellously like scarlet-fever, or severe measles, with a dash of virulent sore throat; or perhaps diphtheria, which to me is merely a sore throat taken in a specifically foul air. I did not get much satisfaction about equine scarlet fever, but plenty of information about the so-called "horse ail," or "horse distemper," or influenza, or pink-eye, or acclimating or seasoning fever, strangles and what not. Still some very experienced horsemen called some things measles, and were inclined to think they were not very far out of the way from scarlet fever, or a hybrid of the two diseases. I knew and was told again and again that the disease or distemper called by all these names and others, breaks out among young, "or green," horses, and seems to rage in some stables more than others, especially in the spring of the year, shortly after their first introduction to city life, when the sale stables are crowded with new comers: caused perhaps by the change from pure air to one contaminated with putrid, ammoniacal and other street, gutter, and stable gases. It is the general opinion that all horses must have an attack of this sort once in their lives, therefore, as all know, a horse that has already had it, is considered acclimated and finds a more ready sale and higher price. Strangles, I was told, and knew, arises from similar causes in young horses from colthood and teething time up to the eighth year, after which it is rare. It is common in the conntry among newly-weaned colts, who are taken from their mothers and from ranging in the fresh air, to be shut up in close stables, or dirty cow, farm, or stable yards, where they inhale a very impure atmosphere, which irritates their throats, eyes and noses.

I also met with cases of typhoid, or septic-pneumonia from the same causes, and became well convinced that some infectious and contagious diseases of the nose, throat, lungs, and other parts were bred anew in filthy stables. I had helped to track down the great horse influenza of 1873, and was well convinced that it was sometimes infectious and contagious. I was especially interested in many cases of so-called "pink eye," attended with discharges from the nose, eyes, sore throat, swelling of the glands of the neck and more or less dropsy, and sometimes by what was called purpura. I suspected that this disease was contagious and a good deal more than a common cold or influenza. I did not then know how to look for the scarlet spots in the nostrils and mouth, nor upon the skin. I found these diseases in private as well as in livery and public stables. In fact some of the former seemed in a worse condition and more offensive to smell than the larger ones, although they appeared well appointed, and in some respects clean. But all the horses had a large quantity of bedding, both by night and day, and extending beyond their heels. Into this all the droppings, liquid and solid went, and was long retained and became very offensive; whereas in stalls, where the horses only had straw under their fore feet by day, the droppings and sweepings were more quickly removed.

I brought the whole matter before the New York Pathological Society, but receiving no encouragement I let the matter drop until after the appearance of Dr. Stickler's well-known article in the *Medical Record* of March 24, 1883.

### Second Attempt to Find the Disease.

I immediately commenced my researches anew and with much better results. I found the homœopathic veterinary surgeon of a large stable on Third Avenue, who was quite familiar with the disease and had had cases not long ago. Also the foreman of a large stable on Sixth Avenue, who had been been familiar with it since 1869, having then been instructed by the late Dr. Copeman, a veterinary surgeon of distinction. In some of the large sales stables hundreds of cases had been seen during the last twenty or thirty years, but were regarded as measles. In the Fourth Avenue stables some doubtful cases had occurred, which they wished very much to have a skilled opinion about, but knew not where to apply for it. It seems that some articles by myself, signed "Medicus," had drawn attention to the subject. I found Professor Bates, of the Columbia Veterinary College, thoroughly learned upon the subject, and he had aided me much by loans of books, journals and details of his own experience, both in horses and other animals. Dr. F. Walton gave me one of the best descriptions of horse scarlet fever that I have yet met with. It is published in the *Sanitarian*. Through the kindness of Dr. Oliver W. Moore, one of the Committmen of the County Medical Society, I have obtained the opinion of Dr. R. W. Finlay, V.S., of 126th Street, Harlem. From observation of 600 cases of so-called "pink-eye" in 1881, he is inclined to think that some, or many of them were scarlet fever in a modified form for the following reason: "There were red, or petechial, or scarlet spots on the visible mucous membrane in some cases; the urine was slightly albuminous on the second day, with slight dropsy about the hind fetlocks; urine still more albuminous on the third day, and petechial spots on the visible mucous membranes still more evident, with tenderness of the glanular structures generally; difficult deglutition; some cough. On the fourth day, extension of the dropsical swellings upwards; some arthritis; temperature 107° to 108°; pulse rapid. The general symptoms were stationary on the fifth and sixth days, but more stiffness and rheumatic and synovial troubles were evident, while the urine became highly albuminous, in some cases tinged with blood and with nephritic colic in other cases. On the 7th day there was slight desquamation, commencing in the ears, and the disease then seemed most communicable or contagious. The improvement was still greater on the ninth day, and by the fourteenth day the majority of the animals were at light work." I have met with other veterinary men who were still more decided in their opinions that all cases of pink-eye were examples of scarlet fever, but I think this is going too far. Dr. Stickler, it is well-known, has succeeded in inoculating two fresh, young and vigorous colts with human scarlatinal virus, and Dr. Fenner, a veterinary surgeon of long experience, pronounced the implanted disease similar to mild cases of scarlet fever in horses which he had seen in his own practice, and of which he had had many. Dr. Lyon also prononnced them cases of scarlatina. Dr. A. N. Bell tells me that Dr. Bailey, of Albany, has met with cases, and quite lately I have come upon the track of the case of the celebrated horse or mare Music that had scarlet fever, in Orange County, the full particulars which I hope soon to receive.

In the meantime I have looked over the whole literature of the subject in the Astor, New York Hospital, and Academy of Medicine Libraries, also in Fleming's Animal Plagues, and Webster's and Basham's work on Epidemics, and in Theophilus Thompson's Annals of Influenza. I am perfectly certain that equine scarlatina has an historical basis which cannot be shaken. In my articles in the *Sanitarian*, April 19th and 26th, May 10th and 31st, June 28th and Oct. 18th, 1883, I intentionally included cases of Angina Maligna and Diphtheria in horses as well as scarlet fever and allied diseases: for the question of diseases in animals communicable to man is by no means limited to scarlet fever. I had intended to review and criticise my own work ere this and will do it soon, as unsparingly as another critic may be inclined to.

### Percivall's Scarlatina, or Rosalia.

As all our knowlege of scarlet fever in horses during the last fifty years dates from Dr. Percivall's articles in the *Veterinarian*, vol. 7, p. 592, London, 1832, I will quote it. It appeared thus:

### SCARLATINA IN HORSES.

BY W. PERCIVALL, V.S.

"Though introducing a new name, I may, perhaps, not be introducing many of the readers of the *Veterinarian* to a disease they have not seen. * * * However, it is one on which our schools and authors have, I believe, hitherto maintained silence. I venture to christen it *scarlatina* and for the reason that in its leading characters it bears more resemblance to that disorder than any other with which I can compare it, and because it seems to answer nearly or quite as well to that appellation as the disease (in man) which is properly so called.

"This affection is commonly ushered in by symptoms of catarrh, for which indeed *it might be mistaken* were these not *in a day or two* succeeded by the appearance of numerous scarlet patches upon the membrane lining the nose, possessing the hue of arterial blood, irregular in size and figure and visible as high up as the membrane can be inspected. These appearances (or blotches) mostly assume the character of (scarlet) petechiæ (or patches), though I have seen them running in streaks. They look first like so many patches of extravasated blood. * * * In passing our finger over these red spots (in the nostrils) nothing like a pimple or pustular elevation of surface is discoverable.

"The skin is everywhere similarly bespotted, at least I infer so from the results of my examination of the body of a horse that died of the disease, whose case I shall annex to this account.

"A mucous discharge continues from the nose. Anasarca is a common attendant. The legs, the sheath and even the belly are occasionally much tumefied.

"The respiration is quickened, but rather indicates pain than embarassment. The pulse is accelerated and beats with force. There exists a great disinclination to move about. The appetite is either wholly lost, or very much impaired.

"Two of my cases recovered and one died. A brown colt was admitted to the hospital with a catarrhal flow from the nose and the schneiderian membrane (was already) everywhere covered with scarlet spots, looking like so many patches of extravasated blood. The lips were prodigiously tumefied and had the same tuberculated chorded feel that farcy would give them. The submaxillary glands of both sides were much enlarged, as well as the lymphatic glands of the breast and thighs. There was no anasarca; the legs were perfectly fine. The respirations were quickened; pulse, 100; no appetite: indisposition to move, and much apparent inability to do so in the hindquarters.

"*Autopsy*:—The skin, from the points from which it was stripped, exhibited precisely the same scarlet spotted aspect which the nasal membrane did during life. There was no ulceration in the nose.

"I (Percivall) shall feel obliged to any reader of the *Veterinarian* who will enlighten me on this subject, for at present I must confess I feel in much obscurity."

In reply Dr. Chapman, of Southampton, reports a case Vol. 8, p. 78, of pharyngitis and laryngitis accompanying scarlatina, marked by scarlet patches on the nostrils and upon the skin, more particularly evident where a blister had been previously applied. The upper lip was much swollen. He says this case may be called scarlatina from its analogy to that disease in the human subject, and he very well recollects a similar one whilst a pupil with his excellent friend and preceptor, Mr. Youatt. In that case there were numerous scarlet patches and anasarcous swellings.

In vol. 13, 1840, there is another case reported by Dr. T. G. Wells, of Whitechapel. There was a discharge from the nose; pulse 75. The horse, five years old, would not move one inch from his place; his lips were swollen, glands enlarged, the nostrils covered with scarlet spots, the chest and abdomen became enlarged from external œdematous swellings. Recovery took place in eight days.

In 1844, vol. 17, p. 539, Dr. I. Morgan reported one case from among numerous examples, with all the above symptoms. About this time Percivall suggested that some of the cases might be *measles*, and Field gave cases which he thought were neither purpura nor simple scarlet fever.

In 1848, vol. 21, p. 615, Dr. Wm. Cox Ashbourne reported a fatal case in a mare that had been in a field among cows which had a similar disease, sometimes called the "vesicular epizootic. There were red or purple spots on the conjunctival, buccal, nasal and vaginal mucous membranes. When the skin was removed it was found covered, like a leopard, with spots, inside and out, from the size of a pea to that of a crown piece. The heart was also spotted.

### Eczema, or Scarlatina.

In 1850 Dr. Haycock, V.S., (see vol. 22, p. 561), introduced a new and, perhaps, disturbing element in the diagnosis and symptomatology of equine scarlatina. He gives the case of a five year old horse with catarrh for five or six days, soreness of the throat, difficulty of swallowing, very severe cough, swelled legs, great swelling of the lower part of the face, particularly of the lips and nostrils, and also of the eyes. The swellings were hot and hard, and there were pustules and vesicles of the size of a pea on the lower parts of the legs, most plentiful in and around the anterior region of the hock-joints and fetlocks.

Professor Williams (see Principles and Practice of Veterinary Medicine, p. 378,) also speaks of an amber-colored serosity oozing from moist patches in scarlatina anginosa, and Professor Robertson, see Text Book of the Practice of Equine Medicine, p. 291, describes a phlyctænoid, or vesicular eruption, with a number of minute vesicles charged with serous fluid which ultimately dessicate and are rubbed, or fall off; and on page 296 he says: "The serous fluid quietly oozes through the skin forming minute vesicles, which rapidly rupture, the contained fluid, hardening into very small tear-like masses." This sometimes occurs in human beings. Gregory (see Lectures on Eruptive fevers, p. 155,) says: "The determination of blood to the skin is occasionally so great that miliary vesicles appear interspersed among the patches of efflorescence, especially upon the breast. This variety has been called *scarlatina varioloides.*

### Catarrh, or Influenza, and Scarlatina, or Measles.

Williams, p. 377, says "scarlatina is usually associated with epizootic catarrh (or influenza) and occurs in animals that have been suffering for some days from that disease. * * * On the third, fourth, or even as late as the sixth day after the commencement of epizootic catarrh the animal is seen to be covered with blotches upon the face, neck, body, extremities, * * * nasal membrane, inside of the lips, on the tongue and throat, etc." This is rather the history of measles in the human being than of scarlet fever, and even Percivall called some of his cases *morbillous*, while Robertson adds force to this suggestion when he states (p. 294-5) that a swollen condition of the eyelids is among the earliest symptoms, together with a cough, and discharge from the nose ; but Gregory says, (p. 162) in some cases in man the voice becomes hoarse and hollow, respiration is performed with a noise like that of strangling, hence the Spanish name *Garotilla.* The throat is clogged with viscid phlegm, and the nostrils pour forth an abundance of acrid sanies followed by excoriation of the lips and mouth.

### Hybrid Disease, or Rosalia.*

A good description of this hybrid disease in human beings is to be found in Aitkens Science and Practice of Medicine, vol. 1, p. 345.

*Definition*: A specific eruptive disease proceeded by and accompanied with fever, watery discharges from the eyes and nose, sneezing and sore throat. The eruption appears on the third and fourth day of the catarrhal symptoms, and consists of crimson spots, blotches, or stigmata, rapidly running into patches of an irregular shape, and of sizes varying from a ten cent to half a dollar, according to the severity of the case. The eruption continues from six to ten days, and terminates by desquamation or peeling off of furfuraceous or branny scales.

---

* John Mason Good, one of our most learned and delightful medical writers, says: Scarlatina is a barbarous and unclassical term that has unaccountably crept into the nomenclature of medicine in place of the original, and more classical, *Rosalia*, which it will be his endeavor to restore. Morton had a mortal aversion to the name scarlatina, and Dr. Haen had nearly as great a dislike to it.

This hybrid disease was first described in England, or rather Scotland, by Dr. Robert Paterson of Leith, in 1840. Hildebrand and Copeland considered it a disease possessing characters common both to measles and scarlet fever, as well as characters peculiarly its own. It is caused by the combined poison of two fevers, with probably a dash of a third, its own.

In the first stage there is fever, cough, like that of true measles, soon followed by itchiness, redness, sensitiveness, and watering of the eyes, frequent sneezing and watery discharge from the nose, headache, rheumatic pains, etc.

In addition sore throat is a most constant symptom, varying from slight to severe, but always one of the most characteristic features of the disease as it is in scarlet fever.

This febrile and catarrhal state lasts three or four days before the eruption appears, which comes suddenly all over the body at once, in its first appearance resembling measles, afterwards scarlet fever, or even petechiæ and purpura.

The sore throat and hoarseness increase, the throat is red and swollen internally, and large secretions of mucus collect there, with constant cough, which is rendered doubly severe and painful from the state of the throat.

The eruption lasts from four to ten days and terminates by desquamation of branny scales like those of measles on the body, but larger on the hands and feet, although never as large as those of scarlet fever.

If we do not call Equine Scarlet Fever, Percivall's Scarlatina, we may call it Good's Rosalia, or Paterson's Hybrid.

### Pink Eye and Scarlet Fever.

I have already alluded to the views of Dr. Finlay, of Harlem, about the coincidence, at least, and perhaps connection of some cases of so-called pink-eye, with scarlet fever. Dr. F. Walton had preceded him in a letter to me, which has already been published in the *Sanitarian*, but the most characteristic parts of which will here be reproduced in view of the great importance of the subject, and in simple justice to him and a proper regard for historical evidence.

Dear Doctor—I have observed equine scarlatina along the water-fronts of this city more especially during the prevalence of the disease, known as "pink eye." This name covers multitudes of ignorances and is applied to several different morbid conditions, and I have thought that some of them were pure epidemics of scarlatina. In the variety of pink eye, to which I have applied the name "equine scarlatina, *the onset is sudden*, the invasion being characterized by much prostration, and a temperature running up rapidly until the fifth or sixth day, and varying from 104° to 106°, even 107°. It is *certainly contagious*, for other animals fall sick in the stables within four days after the first appearance of a case in a previously unaffected building, and I have noticed that those horses which had already had the disease do not succumb to it a second time. *There is an eruption on the skin.* In light-colored animals the skin simply appears congested, but the mucous membranes of the eyes, the nasal passages, the mouth, tongue and throat are covered with a distinctly characteristic *red rash*, generally confluent, often punctate and sometimes blotched.

"There is a free catarrhal secretion from the nose and eyes, but the mucous surfaces of the mouth, tongue and fauces finally peel off, leaving a red, raw looking surface (like the strawberry tongue in scarlet fever.) The fever continues until the fifth or sixth days, then gradually subsides and wholly disappears by the ninth to the eleventh days. The sequelæ appear within ten days to three weeks, and are dropsical swellings generally, with albuminous urine then almost invariably present. But sometimes we find a general rheumatic joint affection (acute articular), which ultimately disappears spontaneously. The attacks are usually not very severe. Sub-acute pleurisy is the worst complication, or sequel. In one case there was decided otitis media, from which the animal became totally deaf.

"In my experience young horses take the disease more frequently than older animals, although the latter are not always exempt. The disease is self-limited and often mild, so that frequently palliative treatment only is required."

In the *London Veterinarian* for September of this year, 1883, I have found a communication on pink eye by the surgeon of Burdett-Coutts, Esq., who says it is *contagious* and generally attended with an *eruption*, especially on the quarters. That crescentic and moniliform baccillæ are found in the blood and secretions, and that inoculation with the blood, tears and nasal mucus taken from the infected animals have reproduced the disease in others. The so-called pink eye was thus first given from an effected horse to a cart horse, from that to guinea pigs, and from them to a pony, so that there can no longer be any doubt about its contagiousness.

### Williams & Robertson's Views on Equine Scarlatina.

These are so peculiar and interesting that a short abstract of them should be given.

Robertson, see Text Book of Equine Medicine, London, 1883, p. 290, says, " he has observed that when a severe visit of these eruptive fevers, viz: measles and scarlet fever has occurred among human beings in any particular (place, town, or) locality that some idiopathic cases of this particular fever (scarlet) would show themselves, and that more than the average number of horses suffering from catarrhal affections would exhibit distinctive features of scarlatina." Again, he says " that it often appears as an accompaniment of some other debilitating affection, such as distemper, influenza, strangles, etc., or when not directly associated with them it seems to have some ill-defined relation to them." This so-called " ill-defined relation " may merely consist in the incubation, in the so termed influenza cases, of the disease for four or six days or more upon the nostrils with the accompanying local irritation; and in the second, the incubation for a like period on the throat may be, and perhaps is, mistaken for strangles, or some other form of sore throat.

On page 291 he says: " We cannot with certainty predict which catarrhal or throat affections will be followed by scarlet fever, save in so far as they are the result of contagion or infection, developed from cases which have exhibited similar complications." He hedges elsewhere on these points, but I must hold him to these almost positive admissions of the communicability of the disease to horses from men, and from horses to other horses. He is not aware that it has been reproduced by inocculation, but we know that Dr. Stickler has succeeded in doing this, and although Dr. Robertson, on p. 293, speaks for the spontaneous generation of scarlet fever in horses, he has already admitted infection, or contagion. Williams (see Principles and Practice of Veterinary Medicine, Wm. Wood & Co., New York, 1879, p. 376,) says he has seen numbers of horses suffering from it, viz: scarlet fever. Again, p. 379: For a number of years he was of the opinion that purpura and scarlatina in horses were one and the same disease, but has had occasion to alter this opinion, as typical cases of both diseases are not uncommon in Edinburgh. He, too, does not think the disorder infectious or contagious; but the grooms have doubtless already had scarlet fever and measles when children, and in most stables only a few horses are purchased at a time, and these only will contract the disease, as all the others have probably already had it. The new comers may go through the disorder under the names of distemper, pink eye, or influenza, and what not. He also says, p. 377, it is usually associated with epizootic catarrh, and the production of such an alteration in the blood as induces scarlet fever is due to defective drainage and ventilation of the stable or to overcrowding, by which the air becomes loaded with decomposing animal matter. Sometimes he even thinks a weak constitution will convert a catarrh into scarlatina. The severity of the attack will depend upon the cleanliness of the stable.

### Scarlatinal Catarrh and Genesis of Scarlet Fever.

In human beings known to have been exposed to scarlet fever, or whom we suspect have the disease before the eruption appears, physicians examine the throat often and carefully, for the malady incubates in or on the mucous membranes, generally of the throat, and assails and penetrates them before it can get into and traverse the blood and appear upon the skin. In measles we examine the eyelids, nostrils and throat. It seems that scarlet fever, as well as measles, in horses appears first on the nostrils, as these are so much larger, sensitive and active in these animals than their throats. In some cases the poison may incubate there for two, four, six, or more days, causing irritation and discharge, as measles does, for four days at least before the eruption appears on the skin. This so-called catarrhal trouble was not mistaken by Percivall for influenza, but has by almost all subsequent English veterinary writers, notably by Williams & Robertson. It would be just as erroneous if human physicians were to say that measles is preceded by ordinary catarrh or cold in the head, or influenza, for four or more days, and that the subsequent measels was developed anew or spontaneously out of the preceding catarrh. It really looks so; but the catarrh is a specific measles catarrh, and so, doubtless, is the so-called scarlatinal-catarrh.

As this so-called catarrhal stage is often a very important point, and has led to more confusion than any other, except the eruptions, I will follow it down to its ultimate bearings.

Surgeon Haycock says: "Usually on the third or fourth, and even as late as the sixth day from the commencement of a nasal, or epidemic catarrh, the animal will be left no worse at night, but in the morning may be found affected in a very peculiar manner. The hair about the neck and legs will be elevated in blotches, while the limbs may be found swollen. The mucous membrane of the nose will have upon it a few scarlet spots of variable size, and there is soreness of the throat: etc." This shows that Haycock, perhaps, did not look for the scarlet patches in the nostrils until after the skin eruption had appeared. As before said, the period of incubation in scarlet fever is about six days, and in horses it sometimes seems to incubate in the nostrils instead of on the throat as in human beings.

Williams, p. 377, says: "Scarlatina is usually associated with epizootic catarrh (probably scarlatinal catarrh) and occurs in animals that have been for some days (some days only) suffering from that disease," and attributes the origin of the disease to defective drainage and ventilation of the stable and overcrowding, by which the air becomes loaded with decomposing animal matters, which poison the blood and produce the scarlatinal eruption after they reach and irritate the skin.

Robertson says (elsewhere): "Scarlatina in the horse presents but a faint analogy to that disease in man and is ordinarily merely the sequel to some other disease, such as influenza, strangles, etc., and seems to have some ill-defined relation to them." We will define this again: When it appears primarily as influenza, it is perhaps the first stage of scarlet fever in the nostrils; when as sore throat, or, perhaps, strangles, it is simply the scarlatinal sore throat antecedent to the eruption. It is very probable that many epidemics of scarlatina are overlooked entirely. Williams says: "A weak (and somewhat depraved) constitution may convert a catarrh into scarlatina, or the severity of an epizootic disease may alter the blood and give origin to scarlatina." Robertson dips still deeper into the genesis of scarlet fever. He says the greater number of animal sufferers from scarlet fever are those which immediately antecedently have passed through some constitutional or debilitating disease. The system is charged with effete and deliterious matter, the result of diseased and excessive tissue changes; there is an unhealthy and vitiated state of the blood, which ultimately appears in the shape of eruptions on the skin and mucous membranes. But he is obliged to modify this attractive theory in some cases and about fall back upon contagion again when he says, p. 293: "Scarlatina is seen in cases of recovery from mild as well as from severe attacks of strangles, catarrh or distemper, and is encountered in young and old animals, and in coarse as well as well-bred horses." In human beings there is an immense number of cases of scarlet fever which we cannot trace to contagion from other human beings. I think that great gaps in this want of proof may be filled by contagion from animals, especially horses, and, perhaps, cats and dogs.

*(To be continued.)*

